# Practices in research, surveillance and control of neglected tropical diseases by One Health approaches: A survey targeting scientists from French-speaking countries

**DOI:** 10.1371/journal.pntd.0009246

**Published:** 2021-03-04

**Authors:** Sophie Molia, Juliette Saillard, Koussai Dellagi, Florence Cliquet, Jean-Mathieu Bart, Brice Rotureau, Patrick Giraudoux, Jean Jannin, Patrice Debré, Philippe Solano

**Affiliations:** 1 CIRAD, UMR ASTRE, Montpellier, France; 2 ASTRE, Univ Montpellier, CIRAD, INRAE, Montpellier, France; 3 INSERM, Paris, France; 4 Institut Pasteur International Network, Institut Pasteur, Paris, France; 5 ANSES, Nancy Laboratory for Rabies and Wildlife, Malzéville, France; 6 INTERTRYP, IRD, CIRAD, Univ Montpellier, Montpellier, France; 7 Trypanosome Transmission Group, Trypanosome Cell Biology Unit, Department of Parasites and Insect Vectors and INSERM U1201, Institut Pasteur, Paris, France; 8 Chrono-environnement Université de Bourgogne Franche-Comté/CNRS, Besançon, France; 9 Société de Pathologie Exotique, Paris, France; Lowell General Hospital, UNITED STATES

## Abstract

One health (OH) approaches have increasingly been used in the last decade in the fight against zoonotic neglected tropical diseases (NTDs). However, descriptions of such collaborations between the human, animal and environmental health sectors are still limited for French-speaking tropical countries. The objective of the current survey was to explore the diversity of OH experiences applied to research, surveillance and control of NTDs by scientists from French-speaking countries, and discuss their constraints and benefits. Six zoonotic NTDs were targeted: echinococcoses, trypanosomiases, leishmaniases, rabies, *Taenia solium* cysticercosis and leptospiroses. Invitations to fill in an online questionnaire were sent to members of francophone networks on NTDs and other tropical diseases. Results from the questionnaire were discussed during an international workshop in October 2019. The vast majority (98%) of the 171 respondents considered OH approaches relevant although only 64% had implemented them. Among respondents with OH experience, 58% had encountered difficulties mainly related to a lack of knowledge, interest and support for OH approaches by funding agencies, policy-makers, communities and researchers. Silos between disciplines and health sectors were still strong at both scientific and operational levels. Benefits were reported by 94% of respondents with OH experience, including increased intellectual stimulation, stronger collaborations, higher impact and cost-efficiency of interventions. Recommendations for OH uptake included advocacy, capacity-building, dedicated funding, and higher communities’ involvement. Improved research coordination by NTD networks, production of combined human-animal health NTD impact indicators, and transversal research projects on diagnostic and reservoirs were also considered essential.

## Author summary

The fight against zoonotic diseases, including NTDs, has greatly benefited from One Health approaches over the last 20 years. The results of this survey show the large attraction these approaches have for scientists working on NTD research, surveillance and control activities in French-speaking tropical countries. However, implementing them is still challenging due to inconsistent political will, insufficient dedicated funding and difficulties in building bridges across multiple sectors and disciplines that each have their own vocabulary, priorities, and ways of conducting research and development projects. There is a significant margin of improvement for One Health uptake, which will be favored by studies on prevalence and economic data integrating impacts at the human, animal and environmental levels, and by studies demonstrating the added value of One Health approaches when they are relevant. Scientific NTD networks have a great role to play in terms of breaking down barriers among sectors and disciplines, promoting exchanges of experiences, and coordinating research to avoid duplication and reinforce synergies. In a world where the poorest populations are also the most heavily affected by climatic disasters, social or political crises, and armed conflicts, NTD scientists from all backgrounds need to better team up to develop and implement more effective and impactful research and interventions.

## Introduction

Neglected tropical diseases (NTDs) are a group of communicable diseases that particularly affect low-income populations in tropical and subtropical areas [1]. Even though they threaten more than a billion people and cost developing economies billions of dollars each year, NTDs have for a long time been overlooked by international and national health programs [2]. This can be explained by several factors including the higher priority given 1) to the “big three” diseases HIV/AIDS, malaria and tuberculosis [2, 3], 2) to emerging/pandemic diseases caused by viruses such as A-H5N1 avian influenza, Nipah, SARS, A-H1N1 influenza, Ebola and now SARS-Cov2 viruses [4–6], and more recently, 3) to non-communicable diseases such as cancer, diabetes and hypertension [7]. Political attention on NTDs has also been difficult to trigger because of the under-reporting or lack of data regarding their respective prevalence/incidence [8,9] and the limits of classic diseases prioritization indexes and tools such as the Disability Adjusted Life Year (DALY) for NTDs [10,11]. Furthermore, NTDs are often clinically confused with other illnesses such as malaria, especially in developing countries where laboratory diagnostic capacity is insufficient [12–15].

Advocacy efforts to tackle NTDs issues have gained momentum in the last decade with the launching of the WHO NTD roadmap in 2012, the World Health Assembly Resolution WHA 66.12 in 2013 and the inclusion of NTDs in the Sustainable Development Goal health targets [16]. Large-scale control programs with the support of pharmaceutical companies committing to donate drugs have enabled considerable progress with several NTDs now heading towards elimination [17]. Integrated control strategies have been promoted to increase the efficiency of interventions. This involves targeting several NTDs (or NTDs and other diseases such as malaria, tuberculosis, HIV) at the same time by the same health workers because they overlap geographically and can be controlled by similar measures such as massive drug administration campaigns or improved water, sanitation and hygiene (WASH) management [16,18].

Neglected zoonotic diseases (NZDs) are also a case for integrated control since they all have animal reservoirs. Among these NZDs, five diseases (echinococcoses, African trypanosomiases, leishmaniases, rabies, and *Tenia solium* cysticercosis) are listed as NTDs in the Resolution WHA66.12 [4]. Their prevention and control are best addressed by using a *One Health* approach [19,20]. One Health refers to a collaborative approach of multiple sectors to design and implement programs and research aimed at improving health for people, animals and the environment [21]. This concept has become increasingly attractive over the past twenty years as a consequence of the multiplication of international health crises due to zoonotic diseases (especially A-H5N1, West Nile, SARS, A-H1N1, A-H7N9, Ebola, Zika and Covid-19 viral infections) causing deaths and major economic losses [22]. It has been endorsed and promoted by international organizations including WHO, the Food and Agriculture Organization of the United Nations (FAO), the World Animal Health Organization (OIE) and the World Bank [22,23].

In Africa, the One Health approach has been put into practice in the last decade for the surveillance, prevention and control of NZDs, especially against rabies. Mass vaccination of dogs had indeed been proven the most cost-effective way for reducing the incidence of human rabies and decreasing the expenses of post-exposure prophylaxis [24–27]. On the African continent, English-speaking countries have paved the way by establishing One Health platforms for infectious zoonotic diseases: among the pioneers, one can cite the Coordinating Office for the Control of Trypanosomiasis in Uganda [28], the Zoonotic Disease Unit in Kenya [29], or the Southern African Centre for Infectious Disease Surveillance [30]. One Health operational research and interventions to control NZDs are also more documented for English-speaking African countries, such as Tanzania [31–37], Uganda [38–40], Zambia [7,41,42], Kenya [43], Ghana [6,44], and South Africa [45], than they are for French-speaking African countries such as Chad [27,46,47], Morocco [48,49], Togo [50] and Burkina Faso [51,52].

The number of French-Speaking people in the world is currently 300 million and projections for 2050 reach 715 million [53]. Following the resolution on NTDs adopted in October 2018 in Erevan by the *Organisation Internationale de la Francophonie* (OIF, International Francophone Organization), the francophone network on NTDs established in 2016 [54] set up a One Health working group to investigate practices in research, surveillance and control of NTDs by One Health approaches in French-speaking tropical countries. The objectives of the current survey were to make an inventory of the activities on NTDs involving a One Health component in French-speaking tropical countries; to discuss the constraints and benefits encountered; and to propose recommendations for improved implementation of One Health approaches in the future.

## Methods

### Ethics statement

The study protocol was designed and implemented in compliance with the ethical rules of AVIESAN (French National Alliance for Life Sciences and Health) South. All data analyzed were anonymized. The study was approved by the Immunology, Inflammation, Infectiology and Microbiology board of AVIESAN South.

### Targeted diseases

In order to define a scope for this survey of One Health practices in the field of NTDs, it was decided to establish a list of specific diseases on which we aimed to collect information. All the five NZDs officially listed as NTDs in the Resolution WHA66.12 were included: echinococcoses, trypanosomiases, leishmaniases, rabies, and *T*. *solium* cysticercosis. We also added one more NZD considered by WHO as having a growing body of evidence for its importance in Public Health and that is leptospirosis [55]. This latter disease was chosen because it is a paradigm for One Health in Africa [12] and because expertise on leptospirosis was common among the members of the francophone network on NTDs.

### Survey steps

The survey consisted of two steps: first an online questionnaire and then a workshop including round tables.

The questionnaire was designed in February 2019 and tested with 10 people in March 2019 to collect information on the perception and practices regarding the One Health approach for research on surveillance and control of the six targeted NTDs. More specifically, information was collected on: 1) the relevance of One Health approaches for NTDs; 2) the personal experience of the respondent with regards to applying a One Health approach for his/her work on NTDs; 3) the type of funding used for One Health NTD projects; 4) the possible difficulties that arose when implementing a One Health approach for NTDs; 5) the possible benefits derived from implementing a One Health approach for NTDs; 6) the possible recommendations the respondent may have for implementing a One Health approach for NTDs; 7) general information on the respondent. The questionnaire (available in S1 Text) included a mix of closed (yes/no) and open questions to which respondents could detail their answers. In order to maximize the response rate, the number of questions was limited to 8 so that less than 20 minutes were required to fill in the entire questionnaire.

The questionnaire was generated and launched online with the open-source LimeSurvey online survey tool and hosted on INSERM (French National Institute of Health and Medical Research) servers in accordance to CNIL (National Commission for Information Technology and Civil Liberties) and RGPD recommendations. Information about the questionnaire (including scope, purposes and guidelines) and its access link were sent by email to all the members of the francophone network on NTDs, with the instructions to forward the message to any other colleagues who would have possibly been interested by the survey. The online questionnaire was accessible in April and May 2019 for a first call of solicitations and in July and August 2019 for a second call. Respondents were free to not fill in any question they did not want to or could not answer and they had the possibility to answer anonymously.

Answers to the questionnaire were analyzed in-depth in September 2019. On 1st-2nd October 2019, a workshop was organized in Marseille, France, gathering 36 members of the francophone network on NTDs and representatives of WHO, AFD (French Development Agency), and pharmaceutical companies working on NTDs. Results of the questionnaire survey were presented and discussed, and three examples of success stories of One Health approaches to tackle NTDs were presented in more details. Two round tables were then organized successively with the 36 participants on how to work together in a One Health approach to better fight against NTDs: one on scientific aspects and one on operational/organizational aspects.

### Data analysis

Questionnaire data were exported from LimeSurvey into an Excel (Microsoft) file. Answers to closed (yes/no) questions were analyzed as percentages with Excel pivot table tool and answers to open questions were analyzed based on a qualitative case study methodology [56] by manually coding answers according to the various emerging themes and topics. Quotes were selected based on their representativeness or their revealing quality.

Notes were taken during the round tables of the workshop and their summary was presented and discussed at the concluding session of the workshop. Participants were asked orally if they agreed with the summary and no objection was raised. Results presented here include this summary, complemented by examples or comments raised by the participants of the workshop.

## Results

### Questionnaire survey

Information about the questionnaire and the url address where to access it was initially sent to the 318 members of the francophone network on NTDs, some of whom then forwarded it to other francophone tropical diseases networks (including EPITROP, Réseau International des Instituts Pasteur, and Groupe d’intervention en santé publique et épidémiologie). We estimated that about 3,000 persons were contacted through this forward and that around 15% of them (450 persons) are working on at least one of the six diseases targeted in our study (echinococcoses, trypanosomiases, leishmaniases, rabies, *T*. *solium* cysticercosis, leptospiroses).

A total of 212 persons accessed the online questionnaire and 171 persons filled it, answering all or only part of the questions. Our response rate was therefore roughly estimated at 22% (171 / [318+450]).

Among the 171 persons who filled the questionnaire, 127 indicated the country where they lived: 72 (57%) lived in France, 14 (11%) in Côte d’Ivoire, 8 (6%) in Niger, 6 (5%) in Guinea, 6 (5%) in Switzerland, 5 (4%) in Burkina Faso, 4 (3%) in Democratic Republic of Congo, 2 (2%) in Algeria, 2 (2%) in Madagascar, 2 (2%) in Belgium, 1 (1%) in Senegal, 1 (1%) in Benin, 1 (1%) in Comoros, 1 (1%) in the United Kingdom, and 1 (1%) in New Zealand.

Among the 171 persons who filled the questionnaire, 116 indicated the type of institution they worked for: 46 (40%) worked for a research institute on human/public health, 18 (15%) for a research institute on agriculture/animal health, 17 (15%) for a university, 13 (11%) for a Ministry of Health, 9 (8%) for a hospital, 7 (6%) for a veterinary school, 4 (3%) for an international organization, and 2 (2%) for a Ministry of Agriculture/Livestock. A total of 71 (61%) persons worked for an institution dealing with human health, a total of 28 (24%) persons worked for an institution dealing with animal health, and the other 17 persons (15%) working for a university were more difficult to classify.

#### Relevance of One Health approaches to fight NTDs

For this first question, the vast majority (98%) of the 170 respondents thought that the One Health approach was relevant for the fight against NTDs (Table 1).

**Table 1:** Answers to the question « Does the One Health approach seem relevant to you for the fight against NTDs?” asked during a 2019 online survey of francophone persons working on NTDs.

Answer	Number of answers (%)
Not at all	0 (0%)
Rather no	1 (1%)
Rather yes	29 (17%)
Yes, definitely	138 (81%)
No opinion/ does not know	2 (1%)
**Total**	**170 (100%)**

Fifty-three persons provided comments on why they thought it was relevant, indicating one or more reasons. The most common reason (46 respondents) was that the targeted NTDs are zoonoses with animals and/or the environment acting as reservoirs or sources of contamination for humans. One Health approaches therefore allowed a better understanding of complex epidemiological cycles with human-animal-environment interactions. The second most common reason (14 respondents) was that it allowed a better fight against diseases by designing more efficient interventions for prevention, mitigation and control. Other reasons were that it improved collaborations and communication between human and animal health sectors (7 respondents) and that it saved operational costs (2 respondents). One respondent stated that the One Health approach was essential to improve diagnostic and prophylactic measures for leptospirosis.

Four respondents tempered their statement that the One Health approach was relevant by saying that it was not adapted to all NTDs and to all contexts. For example:

“In most cases, the One Health approach is relevant. At a minimum, better communication/information exchange between the human and animal health sectors is essential and systematically relevant. Then, depending on the country context and the targeted disease, the degree of One Health collaboration will be more or less easy to implement and the costs (especially time needed to build collaborations) will have to be weighed against the expected benefits.”

#### One Health experiences on NTDs

One-hundred-and-nine (64%) of 170 respondents said they had used a One Health approach for their work on NTDs (Table 2). Eight persons specified they had used a One Health approach for cysticercosis, 9 for echinococcoses, 11 for leishmaniases, 25 for leptospiroses, 22 for rabies, and 27 for trypanosomiases. Detailed description of experiences is available in S2 Text.

**Table 2:** Answers to questions on work experience, funding, difficulties, benefits, and recommendations in implementing a One Health approach for NTDs during a 2019 online survey of francophone persons working on NTDs.

Question	Number of «yes» answers	Number of «no» answers	Total
Have you ever used a One Health approach for your work on NTDs?	109 (64%)	61 (36%)	170 (100%)
For your work on NTDs, have you received funding that was specifically targeted towards One Health projects?	22 (26%)	62 (74%)	84 (100%)
Have you experienced any difficulty when implementing a One Health approach for your work on NTDs?	49 (58%)	35 (42%)	84 (100%)
Have you experienced any benefit when implementing a One Health approach for your work on NTDs?	78 (94%)	5 (6%)	83 (100%)
Do you have recommendations for implementing a One Health approach for NTDs	63 (78%)	18 (22%)	81 (100%)

Some experiences described by participants were common for several of the targeted diseases. Eco-epidemiological studies (assessing the prevalence and studying strains in both humans and animals) were mentioned for all the six targeted diseases. Evaluating intersectoral collaborations and their bottlenecks was performed for cysticercosis, rabies, and trypanosomiases. Knowledge attitude practice surveys on households and dog sampling was done for echinococcoses, leishmaniases and rabies. Studying the ecological and anthropological determinants of dog movements was done for echinococcoses and rabies. Joint human/animal surveillance networks were set up for echinococcoses and leptospiroses. And joint doctor/veterinarian clinical case investigations were performed for leptospirosis and rabies.

Furthermore, for cysticercosis, One Health experiences included: prevalence surveys in humans and pigs before and after a mass praziquantel distribution campaign; joint (by both medical doctors and veterinarians) awareness-raising for health staff and communities; risk mapping based on human and pig prevalence data; joint writing of the cysticercosis national control plan; and joint control measures.

For leishmaniases, One Health experiences included: studying the link between reservoir and transmission; developing diagnosis, treatment and vaccination for dogs; testing the associations between genetic polymorphism of *Leishmania* strains and their virulence in humans; studying the antibody response; and studying the transcriptome of infected macrophages.

For leptospiroses, One Health experiences included: developing new diagnostic and typing tools; identifying reservoirs and their serovars (groups of *Leptopira* strains distinguishable from each other on the basis of antigenicity); comparing pathologies in humans and dogs; studying the virulence of strains; assessing the impact of environmental and biodiversity factors; studying environmental survival of *Leptospira* strains; quantifying occupational risks; testing the interest of animal vaccination to prevent human cases; and setting up public health measures.

For rabies, One Health experiences included: joint risk factor surveys; joint training for human and veterinary health personnel (including role-playing); joint control campaigns (including zoonoses management guide, communication/awareness plan, mass vaccination campaigns); participatory approaches (including a network of village volunteers for dog vaccination and management of stray dog populations); economic and impact evaluation of control strategies (targeting humans and/or dogs).

For trypanosomiases, One Health experiences included: studying the phenomenon of infection tolerance in humans and animals; developing diagnostic tests; searching for immunomodulatory molecules synthesized by trypanosomes; and setting up joint control strategies (including vector control, community awareness-raising on bush clearing, livestock-targeted actions).

#### Types of funding used for One Health NTD projects

Only 22 (26%) of 84 respondents said they had received funding that was specifically targeted towards One Health projects (Table 2). Seven projects were funded by the European Union (3 through DEVCO, 2 through FP6 and FP7, 2 through ERDF), 3 by WHO, 3 by DIM1Health (funds from the Ile-de-France region in France), 1 by AFD (French Development Agency), 1 by COI (Indian Ocean Commission), 1 by ANR (French National Research Agency), 1 by IMT Antwerp and the Belgian Cooperation and Development Directorate, 1 by CNRS (French National Centre for Scientific Research), 1 through French cooperation funds, 1 through American NIH (National Institutes of Health) and 1 by the University of Florida.

#### Difficulties encountered when implementing One Health approaches for NTDs

Forty-nine (58%) of 84 respondents said they had encountered difficulties when implementing a One Health approach for NTDs (Table 2). Fifty-one comments were added to describe these difficulties. Human health and animal health were still commonly considered as separate entities and the One Health approach was not enough recognized or even known and therefore it was difficult to get interest and support for. This was true for all kinds of stakeholders from both the public and private sectors including funding agencies (mentioned by 3 respondents), policy-makers (mentioned by 5), communities (mentioned by 3), and researchers (mentioned by 2).

Building collaborations between sectors (human health and animal health) and actors (MDs, vets, scientists, …) proved difficult for 14 respondents because of differences in priorities, history of conflicts or frictions between people, mutual distrust, ignorance of each other’s constraints, and leadership conflict between (and sometimes within) the Ministry of Health and the Ministry of Agriculture or Livestock.

“As a doctor, we usually have a misguided vision of always wanting to be ahead and take the lead […]. We must always work within the limits of what we know, and recognize the merits of the other. We can’t pretend to know everything.”

Building collaborations between disciplines was also challenging for six respondents. They reported a lack of real multidisciplinary projects beyond the exchange of results or services, a low engagement of clinicians in research and a need to better include humanities in health research. They also considered that building real interdisciplinarity took a long time, and that freeing up that time was hard.

“There is a tendency to take a top-down approach and to consider diseases only as biomedical rather than biosocial or biocultural phenomena.”

“It’s complicated to get people who are very focused on their research on a single bacterium or protein to understand the importance of having an interdisciplinary approach.”

Operational difficulties were reported by 12 respondents in relation to coordination, planning, administrative procedures, tasks division, sharing of infrastructures and resources, surveillance, and timely approvals by ethical committees. Four respondents also reported reluctance with information sharing, sometimes at the request of a higher-up supervisor.

Financial difficulties were reported by 17 respondents, mainly to obtain funding or sufficient means. They also mentioned problems in the management and distribution of budgets, and in procedures for fund release (including deadlines incompatible with field constraints such as the rainy season making roads impassable for instance).

“Despite recent recognition, One Health research projects are still difficult to get funded. Reasons for that include: These very transdisciplinary projects are expensive; The One Health concept is unfortunately often a display (like greenwashing in politics), there are One Health calls for proposals that fund projects that are very far from the real One Health concepts; These One Health concepts are still often misunderstood, or very superficially, by the scientific community.”

Technical difficulties were reported by 8 respondents including having access to wildlife, having access to and information on animal samples from slaughterhouses (because of the commercial values of all organs including kidneys, and because of the lack of traceability of animals entering the slaughter chain), having appropriate diagnostic tools for animals, or obtaining enough cases through hospital surveys.

Other difficulties encountered were more related to NTDs than to using a One Health approach for the fight against NTDs, including the lack of interest and funding for NTDs in general, or the lack of interest of the Ministry of Agriculture for diseases of non-livestock animals such as carnivores or rodents.

#### Benefits to implement a One Health approach for NTDs

Seventy-eight (94%) of 83 respondents said they appraised benefits when/after implementing a One Health approach for NTDs (Table 2). Seventy-six comments were added to describe these benefits, the majority (41) of which pertained to increased intellectual stimulation and collaboration. Respondents enjoyed the diversity and complementarity of disciplines, perceptions, knowledge, experiences, skills, tools, and solutions brought together through One Health approaches. It broadened the scope of their knowledge. One Health approaches also reinforced collaborations through improved communication and dialogue between services and disciplines, better knowledge of the habits and constraints of other Ministries, strengthened links among actors, built-up motivation, and shared commitments.

Eighteen respondents said that One Health approaches allowed to increase and refine their understanding of the epidemiology of NTDs, and 39 that it led to improve the efficacy and impact of NTDs control methods and the health of communities and animals.

“The principle of "killing two birds with one stone" is clearly visible in vector control, in an area where both human and animal African trypanosomiases are prevalent, because the effects are felt on both sides”.

Nine respondents found that it brought financial gains through resources pooling, improved organization and broadening of the panel of calls for proposals you could apply to.

Four respondents reported stronger links with field actors through One Health approaches. Improved exchanges with field staff and communities could lead to better appropriation of interventions.

Finally, two respondents thought that One Health approaches brought increased national and international visibility and recognition.

#### Recommendations for implementing a One Health approach for NTDs

Sixty-three (78%) of 81 respondents wrote they had recommendations for implementing a One Health approach for working on NTDs (Table 2). Seventy-four recommendations were specified.

Five respondents said that there should be more communication about One Health approaches, especially as high as at the level of Ministries, and that more funds should be allocated to promote them. In order to convince policy-makers to implement a One Health approach, three respondents said its benefits should be demonstrated by scientific studies, as it has been the case for rabies.

Capacity-building was advocated by four respondents who suggested organizing One Health modules in medical, veterinary and environmental curricula, as well as common training for medical, veterinary and environmental workers. Organizing meetings with colleagues from other countries in order to exchange experiences on One Health was also suggested by three respondents.

In terms of operationalizing One Health approaches, it was recommended to establish maps of all and key stakeholders; to constitute multi-sectorial and multi-disciplinary teams or networks (including ecology, humanities and WASH, which are often overlooked); to involve as early as possible the different Ministries concerned by NTDs (including the Ministries of Education, Environment, Forestry, Water and Sanitation, and Planning when needed); to be patient and to take time to understand the positions of the various sectors and disciplines involved, and to create links and improved relationships; to co-identify the targeted objectives and research questions; to co-identify and level out as much as possible all regulatory, logistical and methodological issues (including creating legislative and structural frameworks when needed); to increase coordination, integration and synergies for field operations; to permanently communicate and share relevant information (including to public authorities, researchers, and communities); to set up follow-up indicators for measurable objectives.

“Inter-sectoral and inter-disciplinary collaborations take time: it is necessary to get to know each other to gain confidence, to learn how to share potentially sensitive information (e.g. to show that there is little surveillance data, to show that the situation has not improved, etc.), to learn how others work and what their constraints are, to learn how to let go of certain prerogatives, to learn how to share often already limited funding. All this co-construction time must be planned to avoid activities being carried out in an emergency and thus disrupting collaboration. Social events (meals, celebrations, etc.) help to create links where there was mistrust.”

Involving local actors (field staff and communities) was also pinpointed as essential: putting human communities back to the heart of the approach within a general picture of sustainable development; developing participatory approaches to support stakeholders from the very beginning of the project; and considering local beliefs, practices and reluctances. Farmers’ associations were perceived as relevant interlocutors.

“Take the time necessary to solicit support from everyone. Base actions on local resources and endogenous knowledge.”

In terms of funding, it was recommended to increase funds dedicated to One Health projects for NTDs (especially for operational research) and to facilitate studies and projects targeting several NTD pathogens at the same time (for example management of stray dogs could benefit to the fight against rabies, echinococcoses and leishmaniases at the same time).

Finally, it was mentioned that One Health should not be an objective per se, but rather a mean to improve the fight against NTDs, and also that there was no “one size fits all” recipe to implement One Health.

“The approach must be thought out for each country according to its organization, its culture and also its human and financial resources.”

### Workshop discussions

The conclusions of the two roundtables dwelling upon how to work together in a One Health approach to better fight NTDs are presented hereafter.

#### General observations

Some specific characteristics of NTDs were highlighted which make the fight against them particularly challenging. They receive little attention and funding despite the number of people they affect (in comparison, Ebola seems to receive disproportionate research funds). The better results you obtain in NTD control (and hence the closer you get to elimination), the more difficult it is to obtain support from the public authorities and funding agencies. NTDs are diseases associated with poverty, which means that the cost of control measures (such as vaccinating dogs, treating animals with trypanocides, building and maintaining latrines) are often unaffordable at both the individual and community levels or judged as non-priority expenses by the populations affected.

#### Operational recommendations

Funding agencies should provide more money for NTDs and in the framework of a long-term engagement. They should stop partitioning funding opportunities between the human, animal and environment sectors, as it has mainly been the case up to now. Instead, they should favor more transversal research and control projects, for example by dedicating calls for proposals to One Health projects. A single funding agency bringing a significant amount of money to a specific One Health topic can cause the others to follow (e.g. snakebite envenoming). In terms of research funding, agencies need to make sure they provide funds for both fundamental and operational research, the latter being often overlooked and under-endowed. Roundtables with/of pharmaceutical companies donating drugs for the fight against NTDs should also include veterinary pharmaceutical companies, the OIE vaccine bank and alliances (such as GALVmed) to obtain vaccines and treatments for animal reservoirs of pathogens causing NTDs when such products exist and are recommended for NTD control.

Political wills rather than willingness are indispensable to set up One Health approaches and can be particularly complex to focus in countries where different decision makers, e.g. the Ministry of Health and the Ministry of Agriculture/Livestock, do not necessarily have neither a history of harmonious collaboration nor common current priorities. Although a true One Health approach for NTDs should ideally embark all stakeholders (including the Ministries of Health, Agriculture, Environment/Water and sanitation, Education, Rural development, etc.), it may be easier to start with a few stakeholders and add new ones as the project develop and scales up, rather than to wait for all stakeholders to be interested and ready to collaborate. It was also recommended to assign one person, a “One Health champion” at least with a recognized expertise and a strong network, to take the responsibility for getting things started and supervising operations. Once a One Health NTD intervention is well established, a change of leadership is possible to make sure that all stakeholders are fairly involved in the governance.

Advocacy for the Ministries of Education to be involved stems from the observations that children like to learn about animals and are quite receptive to knowledge about diseases whose maintenance potentially involves animals. Integrating into primary school curriculum lessons on NTDs, their prevention and control is a good way to raise long-term awareness in communities.

A clear manifestation of the political will to implement an integrated approach for controlling NZDs and other zoonoses is the creation of an inter-ministerial One Health platform, as it has been the case recently in several West African countries following the Ebola epidemic. However, such a platform should just be considered as an institutional tool and is not sufficient to completely ensure the implementation of a whole One Health approach. In total, adequate resources regarding all in one human, logistic and financial aspects need to be available.

Training is a major component when setting up a One Health approach for the fight against NTDs. It is indispensable because the link between human, animal and environmental health is not obvious for the majority of people. It involves the training of staff as well as that of communities. The commonly high turnover among staff needs to be anticipated as well as a dedicated long-term training plan. Furthermore, and as mentioned before, training of children is a particularly interesting option in the case of NTDs. The francophone network on NTDs needs to establish a list of French-speaking options and experts for specific training on One Health, from tailored short courses to certified modules and Masters (including for example the EcoM-ALGER Master degree designed jointly by the Universities of Kinshasa and Franche-Comté). Other training options include regional workshops to exchange experiences or visits of other research units and laboratories (for example through the European TAIEX program).

#### Scientific recommendations

It is important to coordinate research so that research projects are not duplicated. This is especially true in France where NTD research is scattered among numerous universities and research institutes. Networks such as the francophone network on NTDs could help with this coordination and facilitate useful interactions with the various international NTD networks (in Canada, Germany, Japan, Switzerland, the United Kingdom, etc.).

Scientists working on NTDs need to provide more prevalence and incidence estimates to better assess the impact of NTDs on both humans and animals, and provide baseline data for follow-up and evaluation of control methods. Attracting interests for NTDs from funding agencies and decision-makers will be easier if economic impact studies on One Health programs are available. The need for advocacy is particularly strong for the NTDs targeted in the present study because Ministries of Livestock and the OIE often show less interest for diseases that affect species other than livestock. Zoonotic DALYs need to be calculated to better reflect the impact of NTDs, especially on livestock species.

A lot remains to be done in terms of developing reliable diagnostic tools and protocols for all NTDs in both humans and animals, including transversal diagnosis of several NTDs at the same time. Available diagnostic tools need to be evaluated and margin of progress or gaps identified before developing new tools. Access and availability must be secured. Cheap and rapid diagnostic tests with a better specificity are especially needed and their development is likely to be facilitated by increasing interplay between scientists from the public and the private sector (laboratories and industries).

Involvement of the civil society is essential to increase efficiency of NTD projects. Procedures need to be established for collaborations and some flexibility kept to adapt guidelines to local contexts. Research questions that are developed need to address both the objectives set by national and/or international programs and the needs expressed by communities for their welfare (in terms of rural development, health, etc.). For example, a project to control trypanosomiases in animals is more likely to be adopted by livestock farmers if their main preoccupations (that can be totally unrelated to NTDs and pertain to various issues such as foot-and-mouth disease, gastro-intestinal parasitism, fodder availability, epidemics in vaccinated animals) are also addressed to some extent.

Transversal research questions are essential in One Health approaches as they can benefit to the control of several NTDs. For example, since dogs are reservoirs for the different pathogens causing rabies, echinococcoses, leishmaniases and leptospiroses, information collected on their infection status, structure (with or without owner, confined, restricted, stray, etc), demographics, and mobility could be of interest for controlling several NTDs at the same time. Waste management and pest control (including control of dog populations through the development of immunocontraceptive vaccines) are also transversal issues that would need to be considered when designing NTD control campaigns. One Health should push the ambition beyond the routine call for dialog between medical and veterinary sciences and really include relevant concepts of other fields of science and application like functional and evolutionary ecology, landscape and wildlife management, etc. Humanities also have a lot to bring for more accurately grasping the context, perceptions and levers to get populations more involved in health and NTD management (for example by identifying how to promote responsible dog ownership). Additionally, models for elimination or control campaigns will have to be developed considering the combined repercussions on several NTDs (for example mass drug administration has effects on schistosomiasis, onchocerciasis, lymphatic filariasis, soil-transmitted helminths, but also on cysticercosis).

Finally, researchers from all disciplines should step out of their comfort zone and communicate more extensively together as well as with health authorities and funders. They need to write more policy briefs, advocate NTDs at parliaments and other politic arena, invite decision-makers to scientific meetings and participate to meetings involving high-level health officers (for example meetings of chief veterinary officers at the OIE or in regional networks such as REMESA).

## Discussion

The objectives of the current survey were to make an inventory of the research, surveillance and control activities involving a One Health approach for NTDs in French-speaking tropical countries; to discuss the constraints and benefits encountered; and to propose recommendations for improved implementation of One Health approaches in the future.

To the best of our knowledge, this is the first study of this type and the number of respondents we obtained (171) allowed us to have an estimated 22% response rate, in the line of previous internet-based surveys of health professionals [57]. We only organized two calls of solicitations asking people to fill in the questionnaire and the response rate may have been improved by organizing additional waves [58]. However, our objective was to collect all responses by the end of August 2019 so that the results of the internet survey would be available for the workshop organized in Marseille in October 2019 and a larger than 20% response rate appeared satisfactory to us.

As for other web-based surveys, issues of coverage, non-response and representativity have to be kept in mind when interpreting results. The exact number of researchers and health professionals working on NTDs in French-speaking tropical countries is unknown. The representativity of our survey was therefore difficult to assess, but assuming that 57% of respondents were located in France, it can be hypothesized that it was more representative of French scientists working in French-speaking tropical countries than of francophone scientists working in French-speaking tropical countries. Additionally, our results are more representative of One Health experiences on research than on control/surveillance given that 55% of the respondents worked in research institutions and 15% in universities. Even though research and control/surveillance activities can be jointly implemented [59], it would have been valuable to stratify our analysis based on the distinction between them. Further similar surveys should therefore ask respondents to make this distinction and to provide additional information on their background (e.g. veterinarians, human health professionals, entomologists, environmentalists, etc) for more detailed analyses. Moreover, efforts should be made to enroll more policy-makers and staff of health authorities into the francophone network on NTDs to increase the feedback from persons in charge of surveillance and control. Nevertheless, our aim was more to capture a snapshot of the diversity of One Health experiences applied to NTDs, as well as their perceived benefits and difficulties, rather than to have an exhaustive and representative survey sample.

Another limitation of the survey is the fact that there is no single commonly accepted definition of the One Health concept. Different people may have different interpretation of what One Health entails and the lines between One Health and similar concepts (Ecohealth, Planetary Health, Global Health) are not clear cut [6,60]. Some experiences described by certain respondents as falling under the One Health approach may therefore be ranked low on “One-Healthness” by recent One Health scoring tools [61]. We purposely did not give a specific definition of what could be classified as a One Health project in the introduction of our questionnaire because our goal was to reflect the diversity of One Health perceptions among professionals working on NTDs.

One of the main findings of the present study is that One Health approaches are largely (98%) considered relevant when it comes to fighting against the zoonotic NTDs targeted in the survey (echinococcoses, trypanosomiases, leishmaniases, rabies, *T*. *solium* cysticercosis and leptospiroses). This perception was mainly explained by the fact that the NTDs we targeted were all zoonoses and that One Health approaches brought a finer understanding of their complex epidemiological cycles, and therefore more efficient and comprehensive control methods. The necessary involvement of Veterinary Public Health actors in NTDs control therefore seemed to be commonly accepted, and this is likely the result of communication efforts implemented by international organizations such as WHO, OIE, and FAO [4]. However a huge gap is still to fill with regard to the inclusion of a critical expertise in the fields of ecology, wildlife population dynamics, socio-anthropology and land management [62]. Interestingly, potential financial gain was only cited by two respondents among the reasons why a One Health approach was relevant. The potential financial and logistical added-value of One Health approaches [63] should therefore be more largely promoted to health officers in charge of NTDs. Advocacy for One Health should be favored by publications establishing that the most cost-effective control methods for reducing the incidence of several NTDs in humans involve concomitant interventions on animals (including vectors), such as vaccination or pour-on insecticide treatment of dogs [64,65].

Only 64% of the respondents had put into practice the One Health approach for NTD research and/or control, showing that there is a margin of improvement for One Health uptake. Given the zoonotic nature of the NTDs targeted in the survey, one could advocate that One Health approaches should be almost systematic, especially since 94% of the respondents experienced benefits from implementing a One Health approach. Several of the benefits encountered were very similar to the reasons why One Health approaches were relevant: better understanding of eco-epidemiological cycles, improved efficacy and impact of the fight against NTDs, financial gains. However, interestingly, the most cited benefits were related to what could be viewed as “personal” benefits: increased intellectual stimulation from discovering and experimenting new perceptions, experiences and tools, and improved collaboration bringing motivation, links and shared commitment. These “human connection” benefits can also be found in responses stating that One health approaches improved relations with stakeholders at the local level, including health staff and communities. As far as we know, this benefit of One Health approaches is hardly ever investigated when assessing One Health studies and would be worth putting more attention into. The importance of the human side of the work cannot be overstated, as evidenced in our survey, but also by Stark et al. (2015) who stressed the need for relaxed and friendly encounters of professionals from different backgrounds [66].

Despite its multiple interests, implementing a One Health approach was considered difficult for 58% of the respondents working on NTDs. One of the main difficulties was the on-going separation between sectors (medical, animal and environmental) and disciplines at the institutional, scientific and funding levels. Issues reported in the present survey have already been experienced before, such as publication silos [67], competition for resources and disease-specific programs [68], contradictory agendas between sectors [6], structural barriers and a lack of communication between Ministries [66], leadership conflicts [28], instrumentalizing social science as a mean to nudge behavioral changes [31,62,69], or fiscal year accounting obligations compelling field operations to take place during unfavorable seasons [32]. Our conclusion is that, despite the more than fifteen years elapsed since the promulgation of the Manhattan principles on “One World, One Health” [70] and the dominant rhetoric on the necessity of One Health approaches [23], the compartmentalization between sectors still holds true.

Political endorsement by the various ministries at stake and the creation of adequate institutional frameworks such as One Health platforms was deemed essential. However, experience from Uganda, Kenya and Nigeria shows that these are not sufficient to sustain One Health approaches [28,40,64]. National financial commitment is a key requirement, as well as clearly assigned responsibilities (regarding leadership, communication, operations but also resource allocation) and engagement at all levels of government offices (from field staff to decision-makers). Collaborations between the ministries of Health and Agriculture/Livestock were common in the survey but the Ministry of Education was never mentioned. Efforts to include education authorities in NTD control programs should be stepped up since promising results in terms of both awareness raising and surveillance have been documented by integrating NTD education into primary school curriculum [71].

Our recommendations to better involve local staff and communities and address their concerns stemmed from the observation that too many NTD control programs were implemented top-down and therefore encountered unsatisfactory acceptance by the civil society. Similar findings have been reported in studies assessing One Health networks [68], and participatory approaches are more and more advocated for a wide array of zoonoses [7,72,73].

In conclusion, scientists from French-speaking countries working on zoonotic NTDs who responded to our survey largely approved One health approaches even though less than two thirds of them had already experienced such approaches. Difficulties were commonly encountered when implementing One health mainly in relation to building bridges across health sectors and disciplines, and obtaining dedicated funding and consistent political support. Advocacy and capacity-building should help multiply One health experiences and the benefits they bring in terms of more impactful and cost-efficient interventions for NTD control and elimination.

## Supporting information

S1 Text Questionnaire used during a 2019 online survey of francophone persons working on NTDs. (DOCX)

S2 Text Answers on “One Health work experience” provided by respondents during a 2019 online survey of francophone persons working on NTDs. (DOCX)
