# Supplementary material for: Practices in research, surveillance and control of neglected tropical diseases by One Health approaches: A survey targeting scientists from French-speaking countries
Source: PLoS Negl Trop Dis. 2021 Mar 4;15(3):e0009246. doi: 10.1371/journal.pntd.0009246 (PMC7963066; doi:10.1371/journal.pntd.0009246)
Supplement: S1 Text — (DOCX) [file pntd.0009246.s001.docx]

**Supporting information 1: Questionnaire used during a 2019 online survey of francophone persons working on NTDs**

The One Health approach in the fight against Neglected Tropical Diseases (NTDs)

The Francophone Network on Neglected Tropical Diseases (RFMTN) has adopted the One Health approach as a working theme for the years 2019-2020.

RFMTN's program of activities on the One Health approach includes:

• An online questionnaire survey on the knowledge and practices of the One Health Approach in the fight against six zoonotic NTDs (cysticercosis, echinococcosis, leishmaniasis, leptospirosis, rabies, African trypanosomiasis);

• A workshop to: 1) discuss the results of the online survey; 2) discuss the practices, difficulties and benefits of implementing the One Health approach; 3) develop recommendations for applying the One Health approach;

• Writing an article presenting the results of the online survey and the workshop.

We would be grateful if you would complete the questionnaire. Participation to this survey is on a voluntary basis and you are free to answer only the questions that you want. All published data will be anonymized.

This questionnaire will take 10 to 20 minutes to fill in depending on the extent of your One Health experience.

Yours sincerely,

Juliette Saillard

*Chargée de Mission*

*Institut Immunologie, inflammation, infectiologie et microbiologie (I3M)*

*Inserm / 101 rue de Tolbiac / 75654 Paris Cedex 13*

*Tél : +33 (0)1 44 23 67 20; Email: juliette.saillard@inserm.fr*

1. Does the One Health approach seem relevant to you in the fight against NTDs?

🞏 No, not at all

🞏 Rather no

🞏 Rather yes

🞏 Yes, definitely

🞏 No opinion/ does not know

Additional comments

*Free text*

1. Have you ever used a One Health approach as part of your work on NTDs?

🞏 No (-> end of the survey, please go to question 8)

🞏 Yes (-> possibility to tick several boxes)

🞏 Related to one/several projects on cysticercosis

🞏 Related to one/several projects on echinococcosis

🞏 Related to one/several projects on leishmaniasis

🞏 Related to one/several projects on leptospirosis

🞏 Related to one/several projects on rabies

🞏 Related to one/several projects on trypanosomiasis

1. (This question must be completed for each disease checked in the previous question) Specify the nature of your work using a One Health approach (e.g. research on comparative prevalence in humans and animals, organization of joint training for doctors and veterinarians, improvement of the surveillance system by linking databases, setting up a prevention or control campaign involving several ministries, etc.).
2. To complete your work related to this One Health approach, have you received funding that was specifically targeted towards One Health projects?

🞏 No

🞏 Yes (-> if this box is checked, please specify the nature of the funds)

*Free text*

1. Have you experienced any difficulties when implementing the One Health approach?

🞏 No

🞏 Yes (-> if this box is checked, please specify which difficulties)

1. Did you notice any benefit when implementing the One Health approach?

🞏 No

🞏 Yes (-> if this box is checked, please specify which benefits)

1. Based on your experience, do you have any recommendations for someone who would like to implement the One Health approach in the fight against NTDs?

🞏 No

🞏 Yes (-> if this box is checked, please specify which recommendations)

1. General Information

- Last name : ________________________________________________
- First name : _______________________________________________
- Institution : _______________________________________________
- Country : ____________________________________________________
- Email : ________________________________________

We thank you for your participation.
