# Supplementary material for: Practices in research, surveillance and control of neglected tropical diseases by One Health approaches: A survey targeting scientists from French-speaking countries
Source: PLoS Negl Trop Dis. 2021 Mar 4;15(3):e0009246. doi: 10.1371/journal.pntd.0009246 (PMC7963066; doi:10.1371/journal.pntd.0009246)
Supplement: S2 Text — (DOCX) [file pntd.0009246.s002.docx]

**Supporting information 2: Answers on “One Health work experience” provided by respondents during a 2019 online survey of francophone persons working on NTDs**

| **Disease** | **One Health work experience** |
| --- | --- |
| cysticercosis | eco-epidemiological studies in humans and animals |
| cysticercosis | descriptive and analytical epidemiology (identification of risk factors), risk mapping, awareness and control measures, both in humans and animals |
| cysticercosis | Intersectoral collaboration for national control plan |
| cysticercosis/rabies/trypanosomoses | Appreciate intersectoral collaboration and identify blocking factors |
| cysticercosis | Study of the prevalence of human tapeworm/cysticercosis and porcine cysticercosis before and after mass treatment against tapeworm. |
| cysticercosis | Research and awareness raising work at the scale of village communities in Southern Laos, involving stakeholders (local, provincial and national) from the sectors of animal health, human public health, environment, education |
| cysticercosis/echinococcoses | Studies of the perceptions, practices and knowledge of livestock farmers and communities |
| echinococcoses | eco-epidemiological studies in humans and animals (sheep, dogs, camels) |
| echinococcoses | monitoring network |
| echinococcoses | Community Knowledge and Perceptions, Morocco |
| echinococcoses | Understanding the ecological and anthropological determinants of dog movement and contact in Southeast Asia |
| echinococcoses/leishmaniases/rabies | Household CAP survey + samples from dogs |
| leishmaniases | comparative human/animal prevalence |
| leishmaniases | identification of parasites, vectors and reservoirs |
| leishmaniases | Transmission tank link |
| leishmaniases | research on diagnosis/treatment/vaccine for dogs |
| leishmaniases | research on associations between genetic polymorphism of leishmanias, pathogen complexes and virulence in humans |
| leishmaniases | immunological studies of antibody response and cellular studies in visceral and cutaneous leishmaniasis, transcriptome studies of infected human macrophages. |
| leptospiroses | Identification of tanks |
| leptospiroses | Study of the environmental survival of pathogenic leptospires, the persistence of strains in reservoir hosts. |
| leptospiroses | Search for virulence factors in pathogenic leptospires using genetic and genomic approaches. |
| leptospiroses | Comparative human/animal prevalence |
| leptospiroses | Study of serovars in animal/human / genetic characterization / comparison of isolates from different species (including humans) |
| leptospiroses | Investigation of joint human-animal clinical cases / surveillance |
| leptospiroses | Impact of environmental factors and biodiversity |
| leptospiroses | Risks of transmission of leptospirosis to humans from an animal reservoir (e.g.: market gardening, farms, sewage workers, slaughterers...) / sociological surveys |
| leptospiroses | Investigation of grouped cases of Leptospirosis related to the practice of kayaking/ risk of exposure of people in a context of aquatic activities. |
| leptospiroses | Comparative pathology man dog |
| leptospiroses | Interests and limitations of animal vaccination to prevent human cases |
| leptospiroses | Development of new diagnostic and typing tools. |
| leptospiroses | Collaboration between veterinarians, physicians, biologists and environmental health in the fight against leptospirosis: multidisciplinary investigations, proposing public health measures to reduce the risks of infection, raising awareness, etc. |
| rabies | Joint case investigation / surveillance |
| rabies | Phylogenetic and phylodynamic analysis of strains |
| rabies | Joint risk factor survey |
| rabies | Understanding the ecological and anthropological determinants of dog movement and contact |
| rabies | Joint CAP Survey |
| rabies | Joint training for human and veterinary health service personnel |
| rabies | Collaboration between veterinarians and doctors for the fight against |
| rabies | Design of the National Guide for the Management of Diseases with Epidemic Potential and Priority Zoonoses |
| rabies | Joint communication/awareness plan |
| rabies | Experimental studies of DNA vaccine preparations |
| rabies | Mass vaccination campaign for dogs |
| rabies | Initiation of a network of village volunteers in the vaccination of dogs and the management of stray dog populations. |
| rabies | Assess levels of intersectoral collaboration in the fight, identify blocking factors, propose an operational plan/ Evaluation of a national control, prevention and surveillance network |
| rabies | Economic evaluation of control strategies (targeting humans and/or dogs) |
| rabies | Impact of mass vaccination campaigns for dogs on rabies incidence |
| rabies | Participatory approach |
| rabies | Development of a role-playing game |
| trypanosomoses | Study of the distribution of tsetse flies and trypanosomes (human, animal), and of the factors impacting them (environmental transformations generated by population growth and climate change, etc.) |
| trypanosomoses | Study of animal reservoirs (domestic and wild) |
| trypanosomoses | Study of the phenomena of tolerance to infection in humans and animals. |
| trypanosomoses | Development of diagnostic tests |
| trypanosomoses | Search for immunomodulatory molecules synthesized by trypanosomes |
| trypanosomoses | Coordination of partners from different sectors |
| trypanosomoses | Joint control strategies: anti-vectorial control, community sensitization on bush clearing, actions targeting livestock (chemoprophylaxis, curative treatment) |
